# Exploring wealth-related inequalities in maternal and child health coverage in Latin America and the Caribbean

**DOI:** 10.1186/s12889-020-10127-3

**Published:** 2021-01-10

**Authors:** Manuel Colomé-Hidalgo, Juan Donado Campos, Ángel Gil de Miguel

**Affiliations:** 1 Instituto Tecnológico de Santo Domingo, Universidad Rey Juan Carlos, Madrid, Spain; 2 Universidad Autónoma de Madrid, Madrid, Spain

**Keywords:** Maternal and child health, Health inequalities, Socioeconomic factors, Latin America, Caribbean region

## Abstract

**Background:**

Maternal and child health have shown important advances in the world in recent years. However, national averages indicators hide large inequalities in access and quality of care in population subgroups. We explore wealth-related inequalities affecting health coverage and interventions in reproductive, maternal, newborn, and child health in Latin America and the Caribbean.

**Methods:**

We analyzed representative national surveys from 15 countries conducted between 2001 and 2016. We estimated maternal-child health coverage gaps using the Composite Coverage Index – a weighted average of interventions that include family planning, maternal and newborn care, immunizations, and treatment of sick children. We measured absolute and relative inequality to assess gaps by wealth quintile. Pearson’s correlation coefficient was used to test the association between the coverage gap and population attributable risk.

**Results:**

The Composite Coverage Index showed patterns of inequality favoring the wealthiest subgroups. In eight countries the national coverage was higher than the global median (78.4%; 95% CI: 73.1–83.6) and increased significantly as inequality decreased (Pearson r = 0.9; *p* < 0.01).

**Conclusions:**

There are substantial inequalities between socioeconomic groups. Reducing inequalities will improve coverage indicators for women and children. Additional health policies, programs, and practices are required to promote equity.

**Supplementary Information:**

The online version contains supplementary material available at 10.1186/s12889-020-10127-3.

## Background

Reproductive, Maternal, Newborn, and Child Health (RMNCH) has been a global health policy priority for the past decade [1]. The Millennium Development Goals (MDGs) contributed enormously to the health of women and children, managing to reduce maternal and under-5 years’ old mortality and improved other indicators such as access to contraceptives, skilled attendance at childbirth, and measles vaccination [2]. Despite the progress, most regions did not reach the proposed goals, showing uneven progress that has left gaps between countries, especially in Latin America and the Caribbean (ALC) [3, 4].

The 2030 agenda for Sustainable Development Goals (SDGs) broadens the scope of the MDGs, assuming the commitment to leave no one behind. The SDG-3.8 promotes universal health coverage in terms of access to quality healthcare services, medicines, and vaccines for all [5]. More granular analysis of indicators can show whether all subgroups of the population will benefit from national progress or not [6]. Monitoring inequalities allow identifying vulnerable groups and prioritizing interventions in those who need it the most, thus promoting health coverage through equity [7]. We analyzed the Composite Coverage Index (CCI) as an indicator of universal healthcare coverage gaps in women and children. The index combines preventive and curative interventions throughout the continuum of care, family planning, maternal and newborn care, immunization, and treatment of sick children and has been used to monitor SDGs progress [8, 9].

Previous studies have emphasized the wealth-related inequalities between countries implementing the CCI, but only a few have focused on the LAC situation [10–12]. Therefore, the scope of health interventions and the level of improvement needed to narrow the gap needs to be adequately defined. This study explores wealth-related inequalities in RMNCH care coverage and its impact on reducing the gap in the LAC countries between 2001 and 2016.

## Methods

This was a descriptive study based on secondary RMNCH coverage data obtained from the World Health Organization (WHO) Health Equity Assessment Toolkit (HEAT) software version 3.1 [13]. HEAT performs health inequality measures calculations from the WHO Health Equity Monitor Database [14]. The database includes data from Demographic Health Surveys (DHS), Multiple Indicators Cluster Survey (MICS) and Reproductive Health Surveys (RHS). The surveys carried out national representative and standardized interviews with women 15–49 years old. We included 15 of 22 countries with surveys conducted between 2001 and 2016 based on the availability of recent data on the Composite Coverage Index and wealth quintile.

The CCI is a weighted score based on aggregate estimates of eight essential interventions for the continuum of care for women and children, from before pregnancy to delivery, the immediate postnatal period, and childhood [7, 15]. The index is calculated using the formula:

$$ CCI=\frac{1}{4}\left( DFPS+\frac{ANC4+ SBA}{2}+\frac{BCG+2\mathrm{DPT}3+\mathrm{MCV}}{4}+\frac{ORS+ CPNM}{2}\right) $$

where DFPS = satisfied demand for modern family planning methods; ANC4 = prenatal care (at least four visits); SBA = deliveries attended by qualified personnel; BCG = one dose of Bacillus Calmette-Guérin vaccine; DPT3 = three or more doses of diphtheria-tetanus-pertussis vaccine; MCV = at least one dose of measles vaccine; ORS = children with diarrhea receiving oral rehydration therapy and continuous feeding; NSCLC = children with pneumonia symptoms taken to a health center [16].

We calculated CCI’s, mean, median, interquartile range and standard deviation for the region. We analyzed socioeconomic inequality using the wealth index, which is an estimate based on the ownership of selected assets, housing construction materials, and access to basic services. The details of wealth index estimation have been previously described [17]. Households are classified from the poorest (Q1) to the richest (Q5) [18].

To compare patterns of inequality between and within countries, first, we calculated the coverage difference to show the magnitude of absolute inequality (Q5-Q1); second, the coverage ratio to show proportional differences between groups (Q5 / Q1) and third, the ratio of differences between coverages in lower (Q1-Q2) and higher quintiles (Q4-Q5). We calculated the relative concentration index and slope index to describe inequalities in all subgroups. Finally, we use population attributable risk (PAR) to show the possible improvement if the general population hypothetically had the same coverage level as the wealthiest quintile (CCI-Q5). We estimated the PAR percentage (PAR%) to show the proportion of improvement in national coverage if socioeconomic inequality would have been eliminated (PAR / CCI * 100) [19]. We used Pearson correlation to measure the degree of relationship between the CCI and the PAR%. The analyses were performed using Microsoft Excel and HEAT Plus software.

## Results

Supplementary Table 1 shows the average coverage by wealth quintile for each of the maternal and child health interventions. The coverage gap tended to be smaller as the income level improved. National coverage was greater than 78% in all interventions except family planning and treatment of sick children. The greatest inequality occurred in skilled attendance at birth and prenatal care, where the difference between the wealthiest and the poorest was 26.4 and 17.3%, respectively. The difference was relatively smaller in the immunization indicators, where the absolute inequality was more pronounced in the coverage of DTP3 than in BCG and measles. The difference ratio was well over 1.0 for most of the interventions, showing a wide gap to the detriment of the poorest quintile, except in the vaccination against measles.

Table 1 shows the coverage gaps and inequalities by wealth quintiles for each country. The national median was 78.4% (Range: 49.8% [Haiti] – 86.6% [El Salvador]) and from 71% for the poorest quintiles and 82% for the wealthiest. In three countries - Haiti, Bolivia, and Guatemala - wide differences (> 21 percentage points) were observed between the wealthiest and poorest quintiles. Guyana, Costa Rica, and Paraguay were the only countries with the lowest coverage in the wealthiest quintile. Belize, Costa Rica, the Dominican Republic, El Salvador, Guyana, Honduras, Mexico, and Paraguay showed low levels of inequality, where the difference between the wealthiest and poorest quintiles was 10 percentage points or less. Haiti was the country with the highest level of relative inequality, with coverage in the wealthiest quintile that exceeds that of the poorest by a factor of 1.7. The ratio of differences between the lowest and highest quintiles was greater than 1.0 in nine countries, showing a predominant pattern of higher inequality where the wealthiest quintile had disproportionately less coverage than all the other quintiles, led by Colombia. Reducing wealth-related inequality had the potential to narrow the national gap between 1% (Costa Rica) and 27.9% (Haiti). If all countries could reach the median overall coverage for the wealthiest quintile, the gap would decrease by 3.6 percentage points (95% CI: 2.7–7.1).

**Table 1:** Inequality gaps in CCI by wealth quintile, LAC 2001–2016

Countries	Coverage (%) [CI 95%]											Equity measures			
	Survey	Year	National	Q1	Q2	Q3	Q4	Q5	D	R	RD	SII	RCI	PAR	PAR%
Belize	MICS	2015	77.3 [75.4–79.1]	71.2 [69.1–73.2]	77.1 [69.0–73.1]	79.8 [77.9–81.6]	77.6 [75.7–79.4]	81.0 [79.2–82.7]	9.80 [8.4–11.1]	1.1 [0.6–1.5]	1.7	10.0 [8.6–11.3]	2.0 [1.3–2.6]	3.7 [2.7–4.6]	5
Bolivia	DHS	2008	61.7 [60.5–62.8]	49.4 [48.2–50.5]	58.0 [56.8–59.1]	64.1 [62.9–65.2]	68.8 [67.7–69.8]	73.8 [72.7–74.8]	24.4 [2.0–2.7]	1.5 [1.2–1.7]	1.7	29.4 [28.3–30.4]	7.6 [6.9–8.2]	11.0 [10.4–11.5]	18
Colombia	DHS	2010	81.1 [80.5–81.6]	73.8 [73.1–74.4]	81.9 [81.3–82.4]	84.0 [83.4–84.5]	84.4 [83.8–84.9]	84.7 [84.1–85.2]	10.9 [10.4–11.3]	1.1 [0.9–1.2]	27.0	12.2 [11.7–12.6]	2.4 [2.1–2.6]	2.9 [2.7–3.2]	4
Costa Rica	MICS	2011	84.7 [83.0–86.3]	81.4 [79.6–83.1]	88.2 [86.7–89.6]	80.4 [78.6–82.1]	86.0 [84.4–87.5]	85.1 [83.5–86.6]	3.7 [2.8–4.5]	1.0 [0.5–1.4]	−7.6	2.6 [1.8–3.3]	0.5 [0.1–0.8]	0.9 [0.1–0.7]	1
Dominican Republic	MICS	2014	78.4 [77.6–79.1]	74.3 [73.5–75.0]	78.2 [77.4–78.9]	80.5 [79.8–81.1]	78.3 [77.5–79.0]	82.2 [81.5–82.8]	7.9 [7.4–8.3]	1.1 [0.9–1.2]	1.0	8.0 [7.4–8.5]	1.6 [1.3–1.8]	3.5 [3.2–3.8]	4
El Salvador	MICS	2014	86.6 [85.69–87.51]	84.1 [83.1–85.0]	86.5 [85.5–87.4]	87.5 [86.6–88.3]	86.5 [85.5–87.4]	89.1 [88.2–89.9]	5.0 [4.4–5.5]	1.1 [0.8–1.3]	0.9	5.0 [4.4–5.5]	0.9 [0.6–1.1]	2.4 [1.9–2.8]	3
Guatemala	DHS	2014	68.9 [68.0–69.7]	58.7 [57.7–59.6]	64.2 [63.2–65.1]	70.7 [69.8–71.5]	75.9 [75.0–76.7]	80.1 [79.3–80.8]	21.4 [20.6–22.1]	1.4 [1.1–1.6]	1.3	27.0 [26.1–27.8]	6.2 [5.7–6.6]	10.2 [9.8–10.6]	15
Guyana	MICS	2014	73.7 [71.8–75.5]	70.6 [68.6–72.5]	73.3 [71.4–75.1]	71.6 [69.7–73.4]	78.5 [76.7–80.2]	72.7 [70.8–74.5]	2.1 [1.5–2.7]	1.0 [0.5–1.4]	−0.5	4.7 [3.8–5.5]	1.0 [0.5–1.4]	0 [− 0.9–0.9]	0
Haiti	DHS	2016	49.8 [48.5–51.1]	37.9 [36.6–39.1]	43.8 [42.5–45.0]	51.6 [50.3–52.9]	58.3 [57.0–59.5]	65.3 [64.0–66.5]	27.4 [26.2–28.5]	1.7 [1.3–2.0]	0.8	34.0 [32.7–35.2]	10.8 [9.9–11.6]	13.9 [13.3–14.5]	28
Honduras	DHS	2011	79.7 [78.8–80.5]	74.1 [73.2–74.9]	78.8 [77.9–79.6]	81.3 [80.5–82.1]	82.2 [81.4–82.9]	83.6 [82.8–84.3]	9.5 [8.9–10.1]	1.1 [0.8–1.3]	3.4	11.2 [10.5–11.8]	2.2 [1.9–2.5]	3.6 [3.2–4.0]	5
Mexico	MICS	2015	81.3 [80.2–82.3]	79.4 [78.3–80.4]	78.8 [77.6–79.9]	78.6 [77.4–79.7]	85.4 [84.4–86.3]	86.7 [85.7–87.6]	7.3 [6.6–8]	1.1 [0.8–1.3]	−0.5	1.1 [0.8–1.3]	2.1 [1.7–2.4]	4.9 [4.4–5.4]	6
Nicaragua	DHS	2001	75.3 [74.1–76.4]	63.6 [62.3–64.8]	75.9 [74.7–77.0]	79.2 [78.1–80.2]	79.8 [78.7–80.8]	82.2 [81.2–83.2]	18.6 [17.5–19.6]	1.3 [1–1.6]	5.1	20.5 [19.4–21.5]	4.3 [3.7–4.8]	6.1 [5.5–6.6]	8
Panama	MICS	2013	79.0 [77.7–80.2]	65.2 [63.7–66.6]	83.8 [82.6–84.9]	81.0 [79.7–82.2]	89.0 [88.0–89.9]	83.8 [82.6–84.9]	18.6 [17.3–19.8]	1.3 [0.9–1.6]	−3.6	21.2 [19.9–22.4]	4.2 [3.5–4.8]	3.2 [2.6–3.9]	4
Paraguay	MICS	2016	81.5 [80.1–82.8]	78.9 [77.4–80.3]	81.5 [80.1–82.8]	83.3 [81.9–84.6]	82.3 [80.9–83.6]	82.8 [81.4–84.1]	3.9 [3.2–4.5]	1.0 [0.6–1.3]	5.2	4.3 [3.5–5.0]	0.8 [0.4–1.1]	1.0 [0.4–1.7]	1
Peru	DHS	2016	74.3 [73.4–75.1]	65.4 [64.5–66.2]	74.2 [73.3–75.0]	75.2 [74.4–76]	79.0 [78.2–79.7]	80.8 [80.0–81.5]	15.4 [14.7–16.0]	1.2 [1–1.4]	4.9	17.8 [17.0–18.5]	3.8 [3.4–4.1]	5.9 [5.5–6.3]	8
Median			78.4	71.2	78.2	79.8	79.8	82.2	9.8	1.1	1.3	11.2	2.2	3.6	4.5
Mean			75.7	68.2	75.2	76.8	79.5	81.0	12.2	1.2	2.6	13.9	3.3	4.9	7.3
95% CI for the mean			73.1–83.6	64.2–78.1	71.7–84.6	74.7–84.8	75.5–84.0	78.8–85.5	5.3–14.2	0.9–1.2	1.4–6.8	81.4–196.6	16.9–49.0	2.7–7.1	3.1–11.4
Interquartile range			73.9–81.3	51.7–74.3	73.5–81.8	72.5–81.2	77.8–85.2	80.3–84.5	5.6–18.6	1.1–1.3	−0.2-4.5	4.7–21.2	1.0–4.3	2.4–3.7	2.8–5.3
Standard deviation			9.2	12.6	11.3	8.8	7.5	5.9	7.8	0.2	7.3	10.4	2.9	3.9	7.5

Source: Own elaboration based on study data

CI: confidence interval; MICS: Multiple Cluster Indicator Survey; DHS: Demographic Health Survey; D: difference; R: Ratio; RD: Ratio of differences; SII: Slope index of inequality; RCI: Relative concentration index; PAR: Population attributable risk; PAR%: Percentage of population attributable risk

**Fig. 1:**
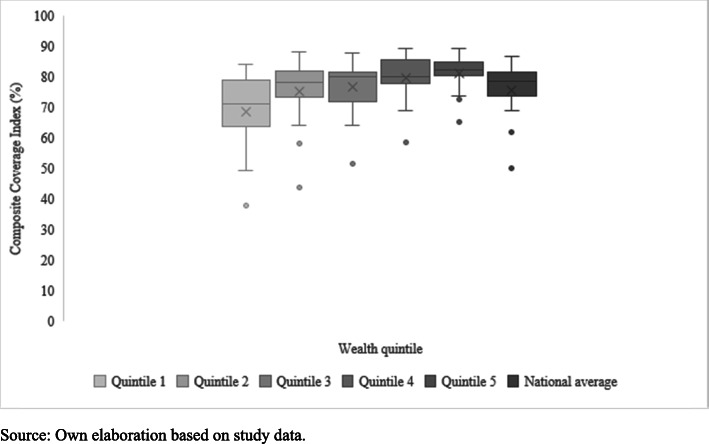
Latest situation of CCI coverage by economic status, LAC 2001–2016. Own elaboration based on study data. ^a^ Dashed lines indicate the median

**Fig. 2:**
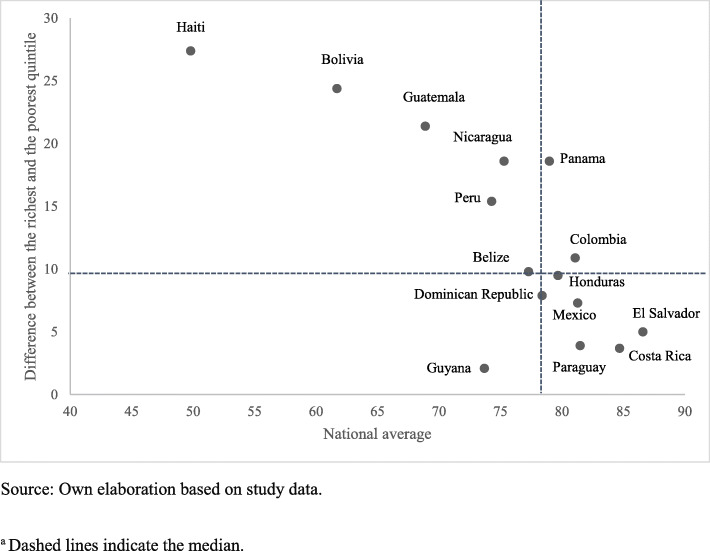
Difference in CCI by country according to wealth quintile, LAC 2001–2016. ^a^. Source: Own elaboration based on study data. ^a^ Dashed lines indicate the median

**Fig. 3:**
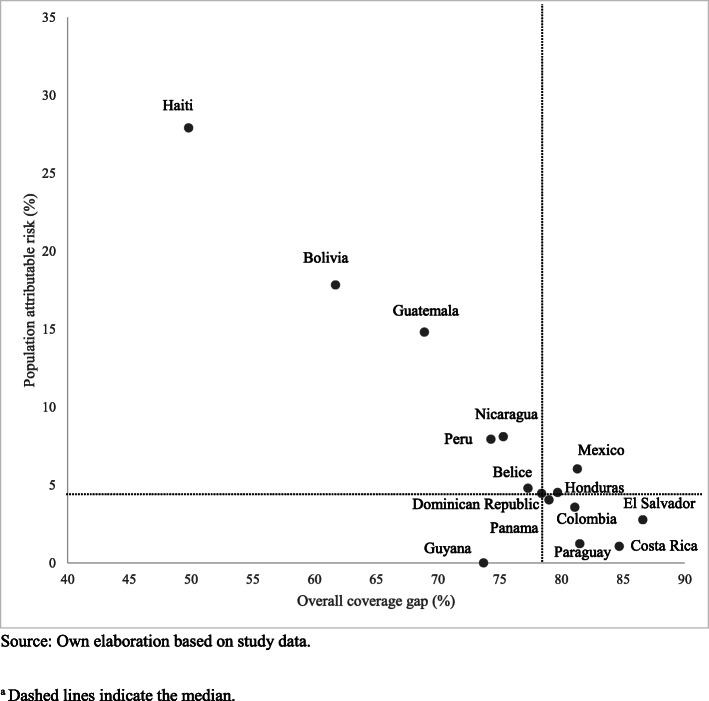
Coverage gap at the national level versus population attributable risk in LAC countries, 2001–2016.^a^. Source: Own elaboration based on study data. ^a^ Dashed lines indicate the median

LAC countries showed a pattern of marginal exclusion in maternal-child health coverage, highlighting the need to address interventions oriented to the most disadvantaged population and also a pattern of higher wealth-related inequality in CCI coverage to the detriment of the poorest quintile (Fig. 1-2). Figure 3 shows the relationship between the CCI gap and PAR% in the study countries. It was observed that healthcare coverage increased significantly as inequality decreased (Pearson r = 0.9; *p* < 0.01). To achieve equality in the distribution of RMNCH interventions, Haiti (27.9%), Guatemala (14.8%) and Bolivia (17.8%) would need to make a greater effort to reduce the ICC gap at their respective levels.

## Discussion

The LAC region has experienced a considerable improvement in maternal and child health post-2015 sustainable development agenda [7]. Despite the progress, it is currently considered the most unequal region in the world, which represents a major challenge for the SDGs [20].

We explore current wealth-related inequalities in RMNCH coverage in 15 LAC countries. Our findings reveal important inequalities in maternal and child health interventions, pointing out that in some groups of the population women and children are lagging.

As shown in this study, essential preventive and curative interventions showed a monotonous pattern with lower levels in the poorest quintile. The inequality gap was greater in interventions that required a functional health system and recurrent interaction with healthcare personnel, except in immunizations. Although approximately 80% of the population benefited from the eight essential interventions, coverage of RMNCH interventions was lower than that in more than half of the poorest countries. Only Costa Rica and El Salvador reached this level in the poorest quintile. The difference between the wealthiest and the poorest was at least 9.8 percentage points in more than half of the countries. Haiti, Bolivia, Guatemala, Peru, and Nicaragua showed lower national coverage and absolute inequality above the regional median. Colombia showed greater inequality of coverage in the top quintiles despite not having a wide gap like other countries. These findings imply the need for health systems that prioritize adequate care to reduce the gaps in women and children from the poorest households [7, 10]. Although the countries of the region have indeed implemented reforms to provide health services without the risk of impoverishment, an approach of social determinants and human rights that considers the dimensions of inequality is still required: income, gender, place of residence and education, among others [21, 22].

Achieving equity represents a much greater challenge for Colombia, Costa Rica, Haiti, Honduras, Mexico, and Panama than for other countries in the region, since they are part of the ten most unequal countries in the world [23]. If wealth-related inequalities were eliminated, most countries could achieve coverage of RMNCH interventions of more than 82%. The relationship between CCI and PAR% suggests that to reduce the gap in coverage of health services, the implementation of policies and programs can be effective in addressing inequalities within each country [11]. Policies should be focused on five areas: (i) development of health infrastructure; (ii) health promotion; (iii) health human resources; (iv) healthcare financing, and (v) quality of care [24–26].

There is a political commitment to understanding inequalities, encompassing efforts to support the monitoring and evaluation of inequities, health policies, and systems. However, the possibilities of achieving the SDG goals will depend on the ability of countries to accelerate and maximize their achievements in well-being [27]. The study, publication and discussion of the determinants of equity in the coverage of interventions and their impact on health contribute to increases in the effectiveness of public policies [28].

This study has several limitations. Coverage estimates are based on reanalyzed data from demographic surveys with a cross-sectional design. The analysis is limited to the availability of recent surveys in each country for latest situation analysis. Because the ICC is a group indicator, HEAT does not provide sufficient data to estimate the standard error using resampling methods [7]. The household ranking of the wealth index may vary by year and country. The described limitations could underestimate the CCI in study countries, particularly utilizing an index based on selected RMNCH health interventions. Despite the limitations, our findings are based on the best method to explore gaps in care coverage between rich and poor [8].

## Conclusions

Overall, our results suggest that women and children from the poorest households in LAC are far from achieving universal health coverage due to inequalities. Our findings show how RMNCH coverage could improve if inequalities were eliminated. Overcoming inequalities will substantially reduce the extreme poverty gap, maternal and child mortality, and promote sustainable development. Future research is needed to monitor inequalities as a critical component tracking the progress of the SDGs so that no one is left behind. We hope that our findings contribute to the design of public policies and strategies to reduce inequalities for women and children in the LAC region.

## Supplementary Information

**Additional file 1: Table S1**. Mean coverage of inequality gaps in interventions by wealth quintile, LAC 2001–2016.

## Data Availability

The datasets used in this article are available in the WHO Health Equity Monitor Database repository at http://apps.who.int/gho/data/node.main.HE-1540?lang=en. Individual data sets are available by the previous request and can be accessed by UNICEF http://mics.unicef.org/ and DHS http://dhsprogram.com/ websites.

## Abbreviations

ALC: Latin America and the Caribbean
ANC4: Prenatal care (at least four visits)
BCG: One dose of Bacillus Calmette-Guérin vaccine
CCI: Composite Coverage Index
CI: Confidence interval
D: Difference
DFPS: Satisfied demand for modern family planning methods
DHS: Demographic Health Survey
DHS: Demographic Health Surveys
DPT: Three or more doses of diphtheria-tetanus-pertussis vaccine
HEAT: Health Equity Assessment Toolkit
MCV: At least one dose of measles vaccine
MDGs: Millennium Development Goals
MICS: Multiple Cluster Indicator Survey
NSCLC: Children with pneumonia symptoms taken to a health center
ORS: Children with diarrhea receiving oral rehydration therapy and continuous feeding
PAR: Population attributable risk
PAR%: Percentage of population attributable risk
R: Ratio
RD: Ratio for differences
RCI: Relative concentration index
RMNCH: Reproductive, Maternal, Newborn, and Child Health
SBA: Deliveries attended by qualified personnel
SD: Standard deviation
SDGs: Sustainable Development Goals
SII: Slope index of inequality
WHO: World Health Organization

## References

[1] Akseer N, Bhatti Z, Rizvi A, Salehi AS, Mashal T, Bhutta ZA (2016). Coverage and inequalities in maternal and child health interventions in Afghanistan. BMC Public Health.

[2] Naciones Unidas. Objetivos de Desarrollo del Milenio. New York; 2015. http://www.un.org/millenniumgoals/2015_MDG_Report/pdf/MDG.2015.rev.(July1).pdf. Accessed 15 May 2020.

[3] Bryce J, Black RE, Victora CG (2013). Millennium development goals 4 and 5: progress and challenges. BMC Med.

[4] Comisión Económica para América Latina y el Caribe. América Latina y el Caribe: una mirada al futuro desde los objectivos de desarrollo del milenio: informe regional de monitoreo de los objectivos de desarrollo de milenio (ODM) en América Latina y el Caribe 2015. Santiago; 2015. https://repositorio.cepal.org/bitstream/handle/11362/38923/S1500709_es.pdf.

[5] United Nations (2016). Resolution a/RES/70/1. Transforming our world: the 2030 agenda for sustainable development.

[6] Barros AJD, Wehrmeister FC, Ferreira LZ, Vidaletti LP, Hosseinpoor AR, Victora CG (2020). Are the poorest poor being left behind? Estimating global inequalities in reproductive, maternal, newborn and child health. BMJ Glob Health.

[7] Wehrmeister FC, Restrepo-Mendez MC, Franca GVA, Victora CG, Barros AJD (2016). Summary indices for monitoring universal coverage in maternal and child health care. Bull World Health Organ.

[8] Countdown to 2030 Collaboration (2018). Tracking progress towards universal coverage for reproductive, maternal, newborn, and child health. ResearchOnline.

[9] Mujica OJ, Moreno CM (2019). From words to action: measuring health inequalities to “leave no one behind”. Pan Am J Public Heal.

[10] Restrepo-Méndez MC, Barros AJD, Requejo J, Durán P, Serpa LAF, França GVA (2015). Progress in reducing inequalities in reproductive, maternal, newborn, and child health in Latin America and the Caribbean: an unfinished agenda. Rev Panam Salud Publica.

[11] Hosseinpoor AR, Victora CG, Bergen N, Barros AJD, Boerma T (2011). Towards universal health coverage: the role of within-country wealth-related inequality in 28 countries in sub-Saharan Africa. Bull World Health Organ.

[12] Marbach M (2008). Mind the gap: equity and trends in coverage of maternal, newborn, and child health services in 54 countdown countries countdown. Res Polit.

[13] Health Equity Assessment Toolkit (HEAT) (2019). Software for exploring and comparing health inequalities in countries. Built-in database edition. Version 3.1.

[14] World Health Organization (2019). Global Health Observatory data repository. Health equity monitor database.

[15] Wehrmeister FC, Barros AJD, Hosseinpoor AR, Boerma T, Victora CG (2020). Measuring universal health coverage in reproductive, maternal, newborn and child health: an update of the composite coverage index.

[16] World Health Organization. Composite coverage index (%). The Global Health Observatory. https://www.who.int/data/gho/indicator-metadata-registry/imr-details/4489. [Accessed 24 Apr 2020].

[17] Rutstein SOJK (2004). The DHS wealth index. DHS comparative reports no. 6. 1st edition.

[18] World Health Organization. Techical notes. Reproductive, maternal, newborn and child health (RMNCH) interventions, combined. New York. www.who.int/gho/indicator_registry/en/. Accessed 25 Apr 2020.

[19] Schneider M, Castillo-Salgado C, Bacallao J, Loyola E, Mujica O, Vidaurre MRA (2002). Métodos de medición de las desigualdades. Rev Panam Salud Pública.

[20] Programa de las Naciones Unidas para el Desarrollo. Más allá del ingreso, más allá de los promedios, más allá del presente: Desigualdades del desarrollo en el siglo XXI. New York; 2019. http://hdr.undp.org/sites/default/files/hdr_2019_overview_-_spanish.pdf.

[21] Etienne CF (2013). Equidad en los sistemas de salud. Rev Panam Salud Publica/Pan Am J Public Heal.

[22] Adegbosin AE, Zhou H, Wang S, Stantic B, Sun J (2019). Systematic review and meta-analysis of the association between dimensions of inequality and a selection of indicators of reproductive, maternal, newborn and child health (RMNCH). J Glob Health.

[23] World Bank (2016). Poverty and shared prosperity: taking on inequality 2016.

[24] Lassi ZS, Salam RA, Das JK, Bhutta ZA (2014). Essential interventions for maternal, newborn and child health: background and methodology. Reprod Health.

[25] Brizuela V, Tunçalp Ö (2017). Global initiatives in maternal and newborn health. Obstet Med.

[26] Bright T, Felix L, Kuper H, Polack S (2017). A systematic review of strategies to increase access to health services among children in low and middle income countries. BMC Health Serv Res.

[27] Bhutta ZA, Chopra M (2016). Devolving countdown to countries: using global resources to support regional and national action. BMC Public Health.

[28] Moucheraud C, Owen H, Singh NS, Ng CK, Requejo J, Lawn JE, et al. Countdown to 2015 country case studies: what have we learned about processes and progress towards MDGs 4 and 5? BMC Public Health. 2016;16 Suppl 2. 10.1186/s12889-016-3401-6. 10.1186/s12889-016-3401-6 PMC5025828 27633919

