# Supplementary material for: Exploring wealth-related inequalities in maternal and child health coverage in Latin America and the Caribbean
Source: BMC Public Health. 2021 Jan 10;21:115. doi: 10.1186/s12889-020-10127-3 (PMC7798299; doi:10.1186/s12889-020-10127-3)
Supplement: Supplementary file 1 — Additional file 1: Table S1. Mean coverage of inequality gaps in interventions by wealth quintile, LAC 2001–2016. [file 12889_2020_10127_MOESM1_ESM.docx]

**Table 1. Mean coverage of inequality gaps in interventions by wealth quintile, LAC 2001-2016.**

| **Indicators** | **Coverage gap by wealth quintile (%) [SD]** | | | | | | **Equity measure** | | |
| --- | --- | --- | --- | --- | --- | --- | --- | --- | --- |
|  | **National** | **Q1** | **Q2** | **Q3** | **Q4** | **Q5** | **D** | **R** | **RD** |
| Satisfied demand for modern family planning methods | 67.2 [19.3] | 63.6 [19.8] | 70.7 [18.5] | 72.6 [16.6] | 75.7 [15.5] | 78.8 [15.0] | 15.2 | 1.2 | 2,3 |
| Prenatal care (at least four visits) | 88.4 [9.0] | 77.7 [14.5] | 85.8 [11.3] | 88.3 [8.2] | 91.7 [5.7] | 95.0 [3.5] | 17.3 | 1.2 | 2,5 |
| Births attended by qualified personnel | 86.7 [15.9] | 71.6 [25.5] | 86.3 [20.5] | 91.9 [14.8] | 95.3 [10.0] | 98.0 [4.6] | 26.4 | 1.4 | 5,4 |
| Tuberculosis vaccination in children under one year of age | 96.1 [4.0] | 94.0 [6.7] | 96.1 [4.2] | 96.4 [4.7] | 97.4 [2.3] | 98.4 [1.5] | 4.4 | 1.0 | 2,1 |
| Vaccination against Diphtheria-tetanus-pertussis in children under one year (three doses) | 84.2 [11.9] | 80.9 [14.4] | 83.8 [13.7] | 84.6 [13.9] | 86.5 [9.5] | 88.0 [8.9] | 7.1 | 1.1 | 1,9 |
| Vaccination against measles in children under one year | 78.1 [11.1] | 77.0 [12.7] | 77.4 [14.0] | 76.3 [13.6] | 80.0 [10.2] | 82.0 [7.9] | 5.0 | 1.1 | 0,2 |
| Children under five with diarrhea receiving oral rehydration therapy and continuous feeding | 53.7 [15.4] | 49.3 [13.7] | 53.7 [19.7] | 54.6 [15.6] | 57.8 [17.6] | 60.0 [14.0] | 10.7 | 1.2 | 2,0 |
| Children under five with pneumonia symptoms taken to a health center | 70.5 [14.0] | 64.7 [16.3] | 68.8 [19.5] | 65.3 [16.0] | 74.9 [17.8] | 77.0 [15.0] | 12.3 | 1.2 | 2,0 |

Source: Own elaboration based on study data.

SD: standard deviation; D: difference; R: Ratio; RD: Ratio for differences.
